# Estimating Leaf Nitrogen Accumulation Considering Vertical Heterogeneity Using Multiangular Unmanned Aerial Vehicle Remote Sensing in Wheat

**DOI:** 10.34133/plantphenomics.0276

**Published:** 2024-12-05

**Authors:** Yuanyuan Pan, Jingyu Li, Jiayi Zhang, Jiaoyang He, Zhihao Zhang, Xia Yao, Tao Cheng, Yan Zhu, Weixing Cao, Yongchao Tian

**Affiliations:** ^1^ National Engineering and Technology Center for Information Agriculture, Engineering and Research Center of Smart Agriculture (Ministry of Education), Key Laboratory for Crop System Analysis and Decision Making (Ministry of Agriculture and Rural Affairs), Jiangsu Key Laboratory for Information Agriculture, Jiangsu Collaborative Innovation Center for Modern Crop Production, Nanjing Agricultural University, Nanjing 210095, China.; ^2^ Jiangsu Academy of Agricultural Sciences Wuxi Branch, Wuxi 214174, China.

## Abstract

The accuracy of leaf nitrogen accumulation (LNA) estimation is often compromised by the vertical heterogeneity of crop nitrogen. In this study, an estimation model of LNA considering vertical heterogeneity of wheat was developed based on unmanned aerial vehicle (UAV) multispectral data and near-ground hyperspectral data, both collected at different view zenith angles (e.g., 0°, −30°, and −45°). Winter wheat plants were evenly divided into 3 layers from top to bottom, and LNA was obtained for the upper, middle, and lower leaf layers, as well as for various combinations of these layers (upper and middle, middle and lower, and the entire canopy, referred to as LNA_Canopy_). The linear regression (LR) and random forest regression (RF) models were constructed to estimate the LNA for each individual leaf layer. Subsequently, models for estimating LNA_Canopy_ that considered the impact of vertical heterogeneity (namely, LR-LNA_Sum_ and RF-LNA_Sum_) were established based on the relationships between LNA_Canopy_ and LNA in different leaf layers. Meanwhile, LNA models that did not consider the effect of vertical heterogeneity (LR-LNA_non_ and RF-LNA_non_) were used for comparative validation. The validation datasets consisted of UAV-simulated data from hyperspectral reflectance and UAV-measured data. Results showed that LNA_Sum_ models had markedly higher accuracy compared to LNA_non_. The optimal scheme for estimating LNA_Canopy_ was the combination of the upper, middle, and lower layers based on the normalized difference red edge index. Among these models, RF-LNA_Sum_ demonstrated higher accuracy than LR-LNA_Sum_, with a validation relative root mean square error of 19.3% and 17.8% for the UAV-measured and simulated dataset, respectively.

## Introduction

Nitrogen is a crucial nutrient for crop growth [1–4]. Effective nitrogen management is vital for achieving high yields, as both nitrogen deficiency and excess can impact crop yield and quality [5–7]. Proverbially, leaf nitrogen content (LNC) reflects leaf nitrogen nutrition information, and dry matter weight represents crop population characteristics [1,8]. Calculated as the product of leaf dry matter weight (LDW) and LNC, leaf nitrogen accumulation (LNA) is a comprehensive parameter that reflects the nitrogen status, photosynthetic production capacity, and vegetation cover status of crops [7,9–11]. Therefore, real-time monitoring of LNA dynamic changes during crop growth period is of great significance for understanding crop population status and accurately managing nitrogen fertilizer application [6,11–13].

Crop growth is a dynamic process of nitrogen turnover, which causes the 4 elements of carbon, hydrogen, oxygen, and nitrogen contained in the biochemical components of crop (such as chlorophyll, nitrogen, cellulose, lignin, etc.) to produce electronic transitions or molecular vibrations under the irradiation of a certain radiation source, forming a spectral difference of reflection and absorption in specific bands [5–7]. Thus, changes in nitrogen status can be captured by the spectral difference in these sensitive bands [14]. Rapid and nondestructive monitoring of crop nitrogen status, including LNA and LNC, using remote sensing technology, has made remarkable progress [10,15,16]. The vegetation indices (VIs) sensitive to leaf area index (LAI) and chlorophyll contents are commonly utilized to estimate crop nitrogen status based on linear or nonlinear empirical relationships [7,15,17]. This method provides the benefits of simplicity and practical convenience in its application [8,18,19]. For example, ratio VIs such as the red edge chlorophyll index (CI_rededge_), green chlorophyll index (CI_green_), pigment-specific simple ratio index (PSSRa), and red edge relative index (RERI_730_), composed of visible and near-infrared bands, have been commonly used to estimate leaf nitrogen concentration (LNC) in crops due to their ability to mitigate saturation effects [20]. Other normalized VIs, including those utilizing near-infrared or red-edge bands, e.g., green normalized different vegetation index (gNDVI), normalized difference red edge index (NDRE), and modified red-edge ratio index (mSR), among others [15,21], as well as multiband VIs such as enhanced vegetation index (EVI), optimal soil adjusted vegetation index (OSAVI), and angular insensitivity vegetation index (AIVI), among others [18,22], have also demonstrated strong stability in estimating crop nitrogen parameters. However, the physiological growth status of crops exhibits considerable variation across different variety types and growing environments, which can lead to limitations in the expansion and stability of empirical models for monitoring nitrogen nutrition [10,18]. Consequently, recent studies have indicated that certain nonlinear machine learning techniques like random forest (RF), support vector machines, and neural networks, among others, are assuming an increasingly crucial role in crop nitrogen monitoring, supplanting traditional VI models [5,19]. Among those methods, the RF algorithm stands out as it can effectively handle datasets with small sample sizes and high-dimensional or multicollinear conditions. Due to its strong robustness, adaptability, and high precision [23,24], the RF algorithm has been recognized as one of the most popular technologies for predicting rice nitrogen nutrition indicators [23–25], potato nitrogen status [26], or estimation of crop nitrogen for regional scale [23]. However, the effect of crop nitrogen vertical heterogeneity on model accuracy has rarely been considered in related studies.

The internal parameters of crops, such as LNC and leaf photosynthetic rate, vary in vertical space due to the uneven light exposure of crops in the vertical direction, coupled with the vertical nitrogen distribution characteristics of the canopy itself [27]. This variability can affect the accuracy and stability of models used to estimate crop growth parameters [6,7,28]. Consequently, many researchers have focused on the vertical distribution of crop nitrogen, chlorophyll, LAI, and other parameters [27,29–31]. For example, Li et al. [28] analyzed the dynamic variation and spectral response characteristics of LNC, LNA, LDW, leaf chlorophyll content, and other parameters with canopy height in different leaf layers of wheat. He et al. [3] analyzed the vertical distribution characteristics of LNC in rice canopies and proposed a new model for estimating the vertical distribution of LNC based on relative canopy height. Li et al. [7] analyzed the vertical distribution of LNC in rapeseed and developed estimation models for LNC in different leaf layers. Wang and Li [32] established a multilayer canopy radiative transfer model (MRTM) to better simulate the relationship between canopy reflectance and the vertical distribution of ear and leaf characteristics in winter wheat.

However, remote sensing technology using a single view zenith angle (VZA) is still limited in providing a comprehensive characterization of crop canopies due to the strong vertical gradient of physiological and biochemical characteristics within the crop canopy [14]. Research has shown that reflectance in crop canopies captured from vertical VZA is primarily affected by leaves in the upper layer [28]. However, crop nitrogen exhibits an inherent attribute of easy transport and is preferentially allocated to the upper leaves with stronger photosynthesis [6,33]. Therefore, when mild nitrogen stress occurs, the lower leaves of crops may undergo premature aging. During moderate nitrogen stress, leaves in the middle and lower layers progressively shift from green to yellow, displaying clear signs of aging, whereas alterations in leaves in the upper layer are seldom observed. Consequently, it proves challenging to promptly assess the nitrogen status of leaves in the middle and lower layers of crops solely through vertical VZA [14,29,34]. Compared with the vertical observation method, multiangular observation provides an additional effective option for timely monitoring of the spectral characteristics and nitrogen status in the middle and lower layers of crop leaves [34–37]. Moreover, prior investigations into the vertical distribution of crop nitrogen have predominantly centered on near-ground hyperspectral remote sensing platforms, with few research conducted on unmanned aerial vehicle (UAV) platforms. Nonetheless, the UAV platform has emerged as the dominant choice for near-ground remote sensing monitoring of farmland in recent years, owing to its high resolution, swift data acquisition, and extensive area coverage [37,38]. Therefore, further research is needed to effectively utilize the vertical heterogeneity of crop distribution to enhance the accuracy of crop nitrogen estimation at the scale of drone-based remote sensing.

In summary, this study aimed to (a) investigate the vertical distribution of nitrogen in different leaf layers of wheat, (b) identify the suitable VZA and optimal VI for estimating LNA in different leaf layers of wheat, and (c) construct and evaluate a new estimation model for wheat LNA considering vertical heterogeneity.

## Materials and Methods

### Experiment design

**Fig. 1.**
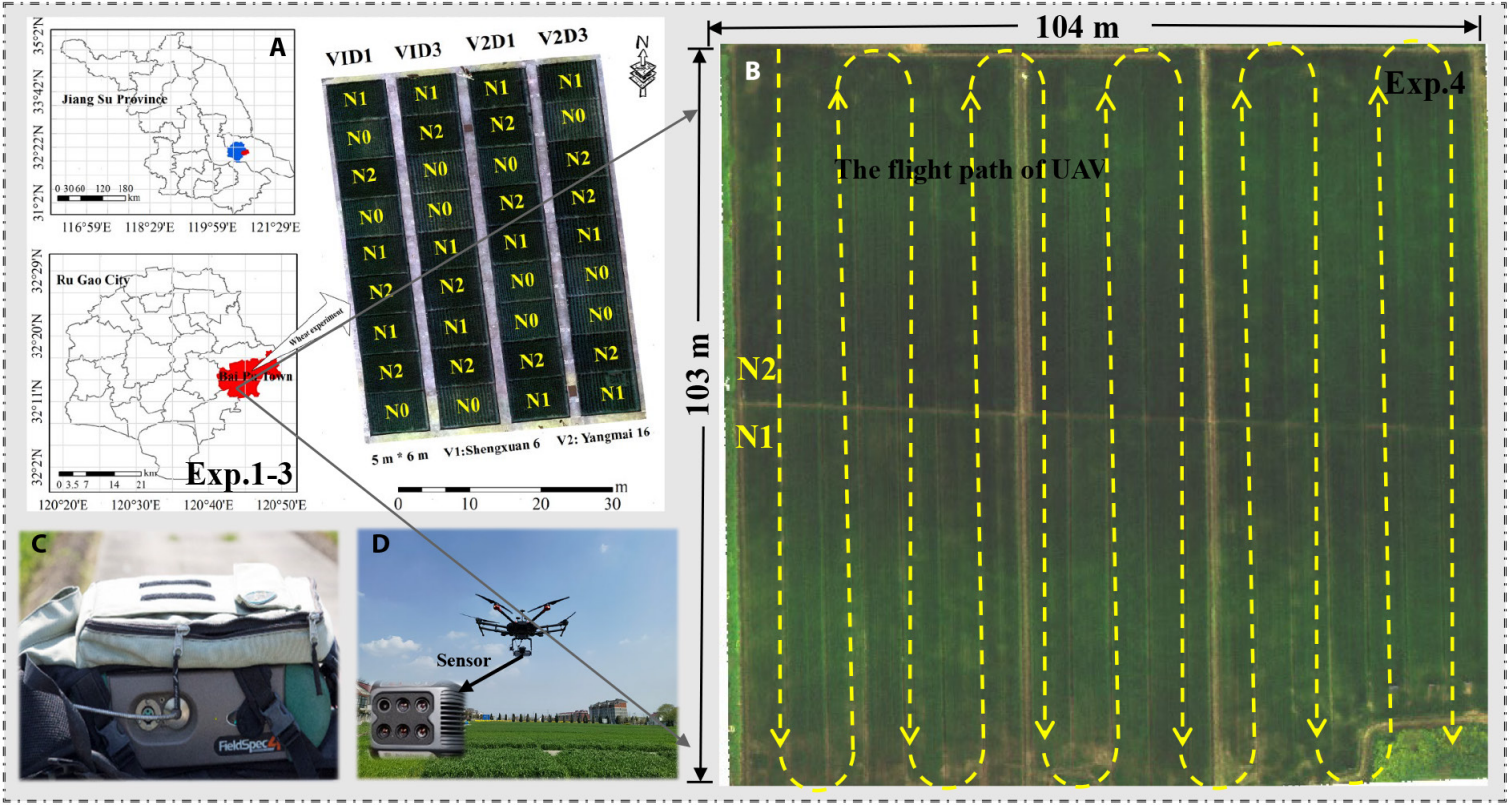
Depiction of the study area and optical sensors: plot experiments (A), field-scale experiments along with the flight path of UAV (B), ASD hyperspectral sensor (C), and airphen multispectral sensor and UAV M600 pro (D). Note: V1 and V2 refer to Shengxuan6 and Yangmai 16, respectively; N0, N1, and N2 correspond to nitrogen application rates of 0, 150, and 300 kg N ha^−1^, respectively; D1 and D3 denote inter-row spaces of 0.25 and 0.40 m, respectively.

Three separate experiments with different treatments were carried out at the National Engineering and Technology Center for Information Agriculture experimental station in Rugao City, Jiangsu Province, China (120°45′E, 32°14′N) (Fig. 1). Experiments 1 and 2 were plot-based studies conducted over 2018 to 2019 and 2020 to 2021, respectively. Each experiment had 36 plots, with an area of 5 m × 6 m per plot. Three repetitions were included in each experiment. Experiment 3, performed in 2020 to 2021, was a field-scale study involving 19 wheat varieties, with the field dimensions being 103 m × 104 m. Details on wheat cultivars, inter-row spaces, and N application rates are shown in Table S1. For all 3 experiments, urea with a nitrogen content of 46% was manually applied as the sole nitrogen fertilizer: 50% before sowing and 50% at the jointing stage. Additionally, 120 kg ha^−1^ of P_2_O_5_ and 135 kg ha^−1^ of potassium fertilizer were applied as base fertilizers. Other field management practices in all experiments adhered to local high-yield field standards. Overall, the various experimental treatments would create a comprehensive dataset with large variations of wheat growing status, which helps to construct more universal crop monitoring models.

### Agronomic parameters acquisition

**Fig. 2.**
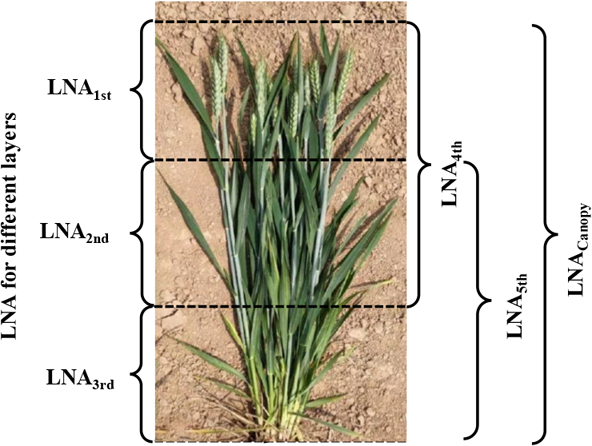
The division of vertical layers within wheat canopy. Note: $\text{LN}A_{1\text{st}}$, $\text{LN}A_{2\text{nd}}$, $\text{LN}A_{3\text{rd}}$, $\text{LN}A_{4\text{th}}$, and $\text{LN}A_{5\text{th}}$ were the leaf nitrogen accumulation (LNA) of the upper, middle, lower, upper–middle, and middle–lower leaf layer, respectively. LNA_Canopy_ was the LNA of the entire canopy.

Agronomic parameters were obtained synchronously with spectral data of wheat canopy. Thirty representative wheat plants were randomly chosen within each plot for measuring plant height, and the mean value was calculated to obtain the canopy average height (H) of the plot. LNA (g/m^2^) was determined by randomly sampling 20 representative wheat plants from each plot. All sampled wheat plants (excluding ears) were categorized into 3 equal layers [3] based on the average height (recorded as first, second, and third from top to bottom, Fig. 2 and Fig. S1), except jointing stage, which is divided into 2 layers due to the shorter plant height. Subsequently, the stems and leaves were separated and dried in the oven for 40 min until a constant weight was achieved. The leaf dry biomass (DW, g) of each layer was then measured using a one-thousandth balance, and the leaf dry weight (LDW, g/m^2^) of each layer was calculated, denoted as LDW_ith_ (*i* = 1, 2, 3, e.g., LDW_1st_, LDW_2nd_, LDW_3rd_). After grinding into powder and weighting, the LNC of leaf samples in each layer (%, denoted as LNC_ith_) was assessed through a C-N element analyzer (vario MACRO cube; Elementar, Hanau, Germany), and LNA in a single leaf layer (denoted as LNA_ith_) was calculated subsequently. At the same time, LNC, LDW, and LNA from the upper–middle and lower–middle layers were called LNC_4th_ and LNC_5th_, LDW_4th_ and LDW_5th_, and LNA_4th_ and LNA_5th_, respectively. The determination processes of LDW_Canopy_ and LNC_Canopy_ were similar to those in the single leaf layer. The formulations for calculating LNA, LDW, and LNC in a single layer, accumulating leaf layer, and canopy were as follows:

$${\mathrm{LNA}}_{\text{ith}}={\mathrm{LNC}}_{\text{ith}}\times {\text{LDW}}_{\text{ith}}$$ (1)

$${\text{LDW}}_{\text{ith}}=N\times {\text{DW}}_{\text{ith}}/n$$ (2)

$$\text{LN}A_{\text{Canopy}}={\text{LNA}}_{1\text{st}}+{\text{LNA}}_{2\text{nd}}+{\text{LNA}}_{3\text{rd}}$$ (3)

$${\text{LDW}}_{\text{Canopy}}=N\times {\text{DW}}_{\text{Canopy}}/n$$ (4)

$${\mathrm{LNA}}_{\text{Canopy}}={\mathrm{LNC}}_{\text{Canopy}}\times {\text{LDW}}_{\text{Canopy}}$$ (5)

$${\text{LNC}}_{4\text{th}}={\text{LNA}}_{4\text{th}}/{\text{LDW}}_{4\text{th}}$$ (6)

$${\text{LNC}}_{5\text{th}}={\text{LNA}}_{5\text{th}}/{\text{LDW}}_{5\text{th}}$$ (7)

where $\text{LN}A_{\text{ith}}$, $\text{LN}C_{\text{ith}}$, and ${\text{LDW}}_{\text{ith}}$ were LNA, LNC, and LDW of layer *i*, respectively. ${\text{DW}}_{\text{ith}}$ was the leaf dry weight of sampling tillers in the *i*th layer. *N* and i were respectively the total number of tillers per square meter of land area (unit: plant/m^2^) and the number of sampling tillers, and *N* was determined by sampling the average number of tillers through 2 rows per meter in each plot. $\text{LN}A_{\text{Canopy}}$ was LNA in the whole wheat leaves.

### Spectral data acquisition

#### Multiangular UAV multispectral reflectance

The multiangular UAV imagery was obtained using the airphen multispectral camera (https://www.hiphen-plant.com/our-solutions/airphen/), mounted on a hexadecopter UAV M600 Pro from DJI technology company, located in Shenzhen, China (Fig. 1D). This camera encompasses 6 spectral wavebands: 450, 530, 570, 675, 730, and 850 nm, each with a 10-nm full width at half peak. The focal length and field of view angle for the 570-nm wavelength are 4.2 nm and 60° × 46°, respectively, while for the other wavelengths, they are 8 mm and 33° × 25°, respectively. The UAV flight was conducted synchronously with destructive plant sampling at key growth stages, including jointing, booting, heading, glowering, forward-filling, and mid-filling stages. Each UAV flight was conducted between 10:00 and 10:30 AM under clear atmospheric conditions, with 14 times of data collection in total. Detailed information on the data collection dates is listed in Table S1.

The sideway and forward overlap rates were set at 94% and 85%, respectively. The UAV flew at a height of 10 m, with a speed of 2.5 m/s, and at various VZAs of −45°, −30°, and 0°. Here, a vertical angle of 0° is defined, while “−” signifies that the sensor’s observation direction aligns with the sun (with a relative azimuth between the sun and sensor of 0°), referred to as the backward direction. The flight path of the UAV is illustrated as Fig. 1B.

#### Multiangular near-ground hyperspectral reflectance

Canopy reflectance data were acquired using a hyperspectral spectrometer, ASD (FieldSpec4 Standard-Res, Analytical Spectral Device, Boulder, CO, USA), affixed to a multiangular observation device. The spectral range, angle of field view, and spectral resolution of ASD were 350 to 2,500 nm a, 25°, and 1 nm, respectively. All canopy reflectance measurements were conducted at a height of 1 m above the wheat canopy on clear days between 10:00 AM and 2:00 PM from early March to early June of the following year after sowing, encompassing the primary growth stages of wheat. The VZAs were −45°, −30°, and 0° in order to keep in touch with multiangular UAV data. The reflectance value at each VZV was determined by averaging 3 measurements taken. Before each measurement, reference plate calibration was performed.

### Preprocessing and analysis of spectral data

Geometric correction and mosaicking of UAV images were performed using Agsoft PhotoScan (Version 1.2.4.2399, Agsoft LLC., Russia) [12]. Subsequently, ENVI 5.3 (Exelis Visual Information Solutions, Boulder, CO, USA) was utilized to extract multiangular reflectance data. The transformation of hyperspectral multiangular data into UAV multispectral data was accomplished by employing the response function (SRF) of airphen sensor (Fig. S2), following the specified formulation [39]:

$$I_{\text{simulated}}\left(\lambda \right)=\frac{\int_{\lambda_{\text{min}}}^{\lambda_{\text{max}}}\;I\left(\lambda_{i}\right)\;*\;\text{SRF}\left(\lambda_{i}\right)d\left(\lambda_{i}\right)}{\int_{\lambda_{\text{min}}}^{\lambda_{\text{max}}}\;\text{SRF}\left(\lambda_{i}\right)d\left(\lambda_{i}\right)}$$ (8)

where $I_{\text{simulated}}\left(\lambda \right)$ was simulated multispectral reflectance, $I\left(\lambda_{i}\right)$ was hyperspectral reflectance data corresponding to band $\lambda_{i}$, and $\text{SRF}\left(\lambda_{i}\right)\;$was the spectral response function of airphen sensor.

### Calculation of VIs

The VIs employed to estimate LNA in this study included normalized VIs (gNDVI, NDRE, and mSR), difference VIs (RD_730_ and RD_850_), ratio VIs (RERI_730_, CI_rededge_, and CI_green_), and VIs with a specific function (AIVI, OSAVI, and EVI), among others. The calculation formulation of each VI is shown in Table S2, and their abilities to estimate LNA have been verified in previous studies [11,14,40].

$$∆\;\text{VI}=\frac{{\text{VI}}_{\theta }-VI_{\text{Origin}}}{VI_{\text{Origin}}}$$ (9)

where VI was reflectance or vegetation indices, $VI_{\theta }$ was VIs at nonvertical observation VZA, and $VI_{\text{Origin}}$was VIs at vertical observation VZA.

### Construction and evaluation of LNA estimation model considering vertical heterogeneity

According to Eq. 3, $\text{LN}A_{\text{Canopy}}$ was the sum of $\text{LN}A_{\text{ith}}$, and $\text{LN}A_{\text{ith}}$ was estimated by linear or RF regression models of VIs (Eq. 10). It is noticeable that the RF model includes 2 main tuning parameters: the number of decision trees (*ntree*) and the number of observations per tree leaf (*mtry*). When *ntree* is adjusted to a sufficiently large value, it mainly affects the execution time rather than the modeling accuracy; thus, ntree was set to 500 according to previous studies [41]. The *mtry* was recommended to set as the square root of input predictor numbers, due to the fact that the RF models constructed in this study were all univariate models; therefore, *mtry* was set to 1. The 10-fold cross-validation of the RF model based on different VZAs and VIs (Table S3) demonstrated its robustness in predicting LNA.

$${\text{LNA}}_{\text{ith}}=f{\left({\text{VI}}_{\text{ith}}\right)}^{\theta }$$ (10)

where *i* was the leaf layer of *i*th and ${\text{LNA}}_{\text{ith}}$ was the LNA of leaf layer *i*. θ was VZA and ${\text{VI}}_{\text{ith}}$ was the VI used to retrieve LNA_ith_. The function *f*(*x*) means the linear or RF regression model.

By combining Eqs. 3 and 10, the estimating model of LNA considering vertical heterogeneity is finally shown as Eq. 11.

$${\text{LNA}}_{\text{Canopy}}=\sum_{1}^{n}k_{n}*f{\left({\text{VI}}_{\text{ith}}\right)}^{\theta }$$ (11)

where the function *f*(*x*) means the linear or RF regression model. The coefficient *k_n_* was obtained from the slope of the linear correlation between LNA_ith_ and LNA_Canopy_. When *n* was 1, representing a single leaf layer, the corresponding LNA was denoted as ${\text{LNA}}_{\text{ith}}$. While *n* was equal to 2, the accumulating upper–middle layer was called LNA_4th_, and the accumulating middle–lower layer was called LNA_5th_. If *n* was equal to 3, it represented the sum of LNA_1st_, LNA_2nd_, and LNA_3rd_, called LNA_Sum_, and at this point, the coefficient *k_n_* of each layer is 1.

**Fig. 3.**
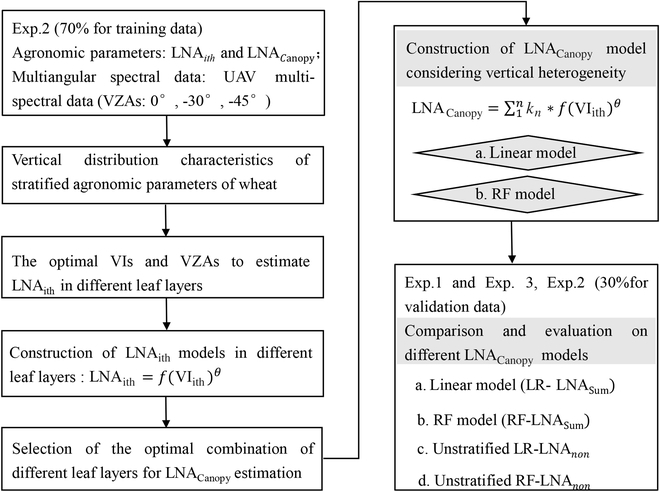
Construction and validation workflow of the $\text{LN}A_{\text{Canopy}}$ model considering vertical heterogeneity of wheat canopy.

The model for estimating LNA was constructed and is shown in Fig. 3. First, the optimal estimation model of LNA_ith_ and the VZA for different leaf layers were determined based on the correlation coefficient (*R*) and relative root mean square error (RRMSE) between various VIs and LNA_ith_. Subsequently, LNA_Canopy_ estimation models were constructed and comprehensively compared based on the retrieval models of LNA_ith_ (*i* = 1, 2, 3, 4, 5) for each leaf layer (Eq. 11), following the identification of optimal combinations of leaf layers to accurately estimate LNA_Canopy_. Notably, among them were LNA_4th_ and LNA_5th_, obtained by accumulating the most appropriate models of the upper–middle and middle–lower layers, respectively, ensuring consideration of vertical heterogeneity in wheat. Then, LNA_Canopy_ estimation models were developed using both linear and RF algorithms. To compare, models of estimating canopy LNA without hierarchical consideration were constructed (noted as LNA_non_). Performance evaluation relied on *R*, *R*^2^, and RRMSE metrics in this study. Moreover, 70% of Experiment 2 data were utilized for developing the LNA_Canopy_ estimation model, with the remaining 30% reserved for validation. Supplementary validation employed data from Experiments 1 and 3.

## Results

### Vertical distribution characteristics of different agronomic indices in wheat canopy

**Fig. 4.**
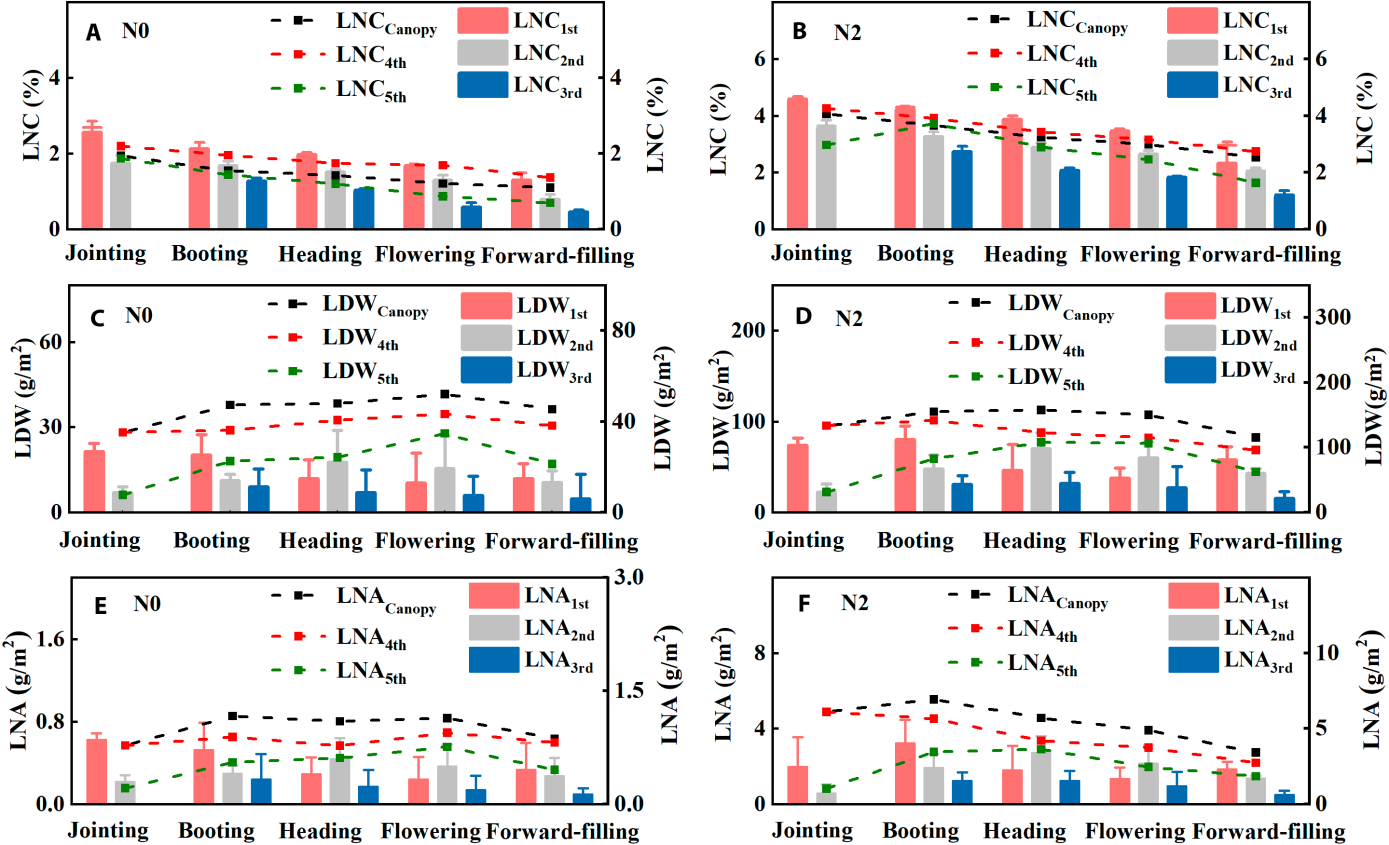
Variation with growth stages of agronomic parameters of different leaf layers: leaf nitrogen content (A and B), leaf dry matter weight (C and D), and leaf nitrogen accumulation (E and F).

The vertical distribution of LNC_ith_, LDW_ith_, and LNA_ith_ in different leaf layers during wheat growth is shown in Fig. 4. LNC_ith_, LDW_ith_, and LNA_ith_ (*i* = 1, 2, 3, 4, 5) exhibited trends consistent with changes of LNC_Canopy_, LDW_Canopy_, and LNA_Canopy_ over the growth periods. LNC gradually decreased with growth stages, and the differences between LNC_Canopy_ and LNC_4th_, LNC_5th_ were small in the early growth stage but increased gradually in the late growth stage, especially under low nitrogen levels. Meanwhile, both LDW and LNA initially increased before declining as the growth stages progressed. LDW_ith_ and LNA_ith_ peaked at the heading stage under high nitrogen levels. However, LDW_ith_ and LNA_ith_ (*i* = 1, 2, 3, 4, 5) reached their maximum values at the anthesis stage under low nitrogen levels. LNC_Canopy_, LDW_Canopy_, and LNA_Canopy_ were higher than LNC_ith_, LDW_ith_, and LNA_ith_ (*i* = 4, 5). LNC_ith_, LDW_ith_, and LNA_ith_ under high nitrogen levels were higher than those under low nitrogen levels, and the differences in LNC_ith_, LDW_ith_, and LNA_ith_ (*i* = 1, 2, 3, 4, 5) among leaf layers were greater than those at low nitrogen levels.

The linear relationship between each LNA_ith_ and LNC_Canopy_ is presented in Table 1. The coefficients (*k_n_*) of LNA_ith_ (*i* = 1, 2, 3) and LNA_Canopy_ in the single leaf layer were 1.36, 2.34, and 3.61, respectively. The coefficients (*k_n_*) of LNA_ith_ (*i* = 4, 5) and LNA_Canopy_ in the cumulative leaf layer were 1.15 and 1.37, respectively. When the number of cumulative layers was 3, the coefficient *k_n_* of each layer was 1.

**Table 1.** Relationship between LNA_ith_ and LNA_Canopy_ (*i* = 1, 2, 3, 4, 5)

Stratified layers	Formula	*R* ^2^
**LNA**_**1st**_	LNA_Canopy_ = 1.36 * LNA_1st_ + 1.12	0.78
**LNA**_**2nd**_	LNA_Canopy_ = 2.34 * LNA_2rd_ + 0.48	0.84
**LNA**_**3rd**_	LNA_Canopy_ = 3.61 * LNA_3rd_ + 0.91	0.73
**LNA**_**4th**_	LNA_Canopy_ = 1.15 * LNA_4th_ + 0.10	0.95
**LNA**_**5th**_	LNA_Canopy_ = 1.37 * LNA_5th_ + 0.95	0.68
**LNA**_**Sum**_	LNA_Canopy_ = LNA_1st_ + LNA_2nd_ + LNA_3rd_	0.97

### Variation characteristics of VI under different VZAs

The relative changes of each VI under different VZAs (0°, −30°, and −45°) are illustrated in Fig. S3. These changes indicated that the relative changes of VIs under high nitrogen levels were smaller than those under low nitrogen levels. Among the VIs, PSSRa, RD_850_, RD_730_, CI_rededge_, and CI_green_ showed obvious relative changes, with average relative changes of 9.51%, 9.39%, 7.48%, 8.35%, and 6.36%, respectively. Following these were OSAVI, EVI, and AIVI, with average relative changes of 6.36%, 5.17%, and 4.93%, respectively. On the other hand, the relative changes of RERI_730_, NDRE, mSR, and gNDVI were generally small, with average relative changes of 3.54%, 2.34%, 1.49%, and 1.29%, respectively.

### The optimal estimation model of LNA in different leaf layers

**Fig. 5.**
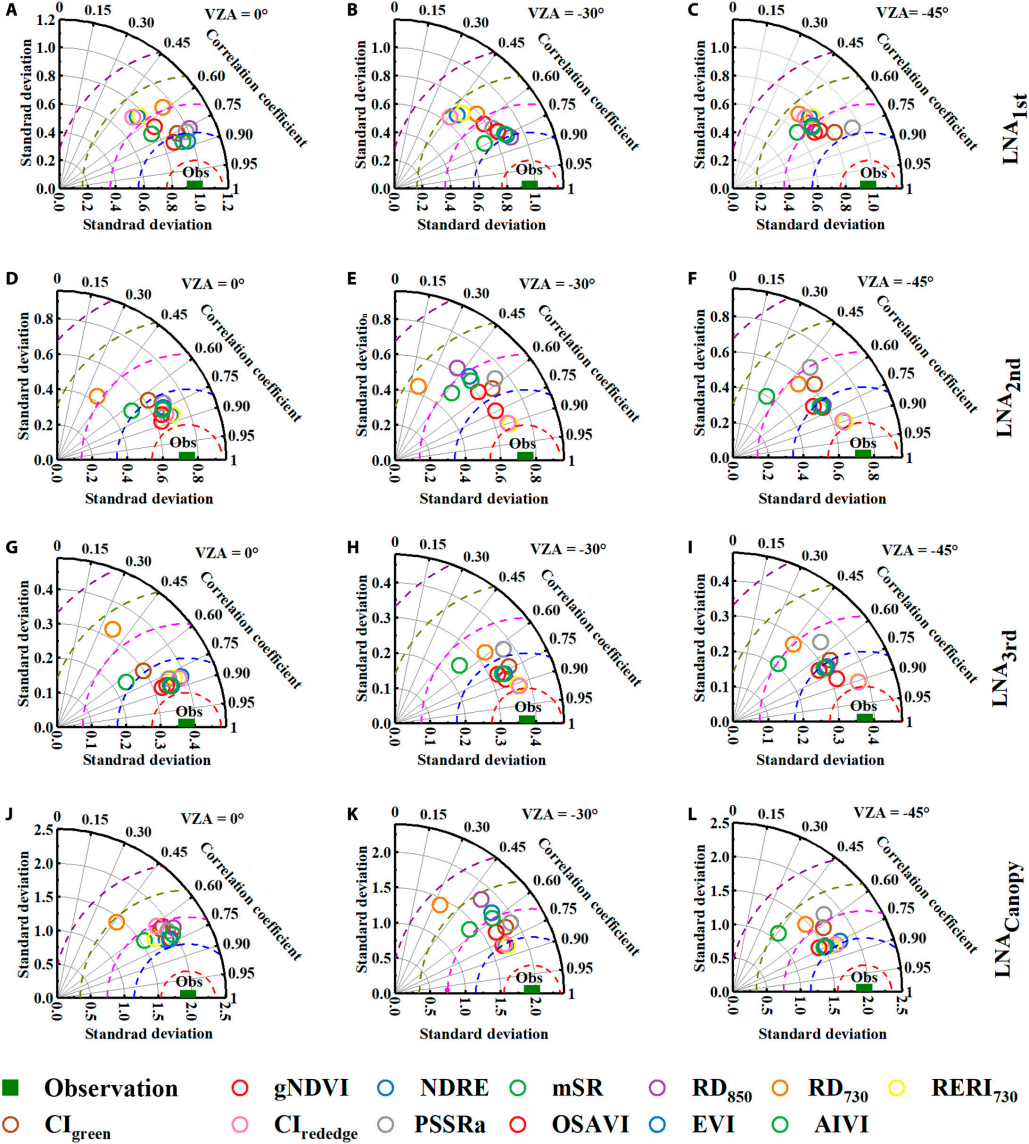
Correlation (*R*) and RRMSE between LNA_ith_ and VIs in different leaf layers and different view zenith angles (VZAs): LNA_1st_ and VIs in different VZAs (A to C); LNA_2nd_ and VIs in different VZAs (D to F);LNA_3rd_ and VIs in different VZAs (G to I);LNA_Canopy_ and VIs in different VZAs (J to L).

The correlation between LNA_ith_ and VIs in different leaf layers and at different VZAs (0°, −30°, and −45°) is shown in Fig. 5. The results indicated that the optimal VI, VZA, correlation coefficient, and RRMSE of LNA_1st_ in the upper leaf layer was EVI, 0°, 0.88, and 18.3%, respectively. For LNA_2nd_ in the middle layer, the optimal VZA was −30°, and the optimal VI, correlation coefficient, and RRMSE were RERI_730_, 0.90, and 18.1%, respectively. Similarly, for LNA_3rd_, the optimal VZA, VI, correlation coefficient, and RRMSE were -45°, NDRE, 0.91, and 18.4%, respectively. The optimal VZAs and VIs for LNA_4th_ and LNA_5th_ were the same as those for LNA_1st_ to LNA_2nd_, and LNA_2nd_ to LNA_3rd_, respectively. The most suitable VZA of LNA_Canopy_ was −30°, with RERI_730_ as optimal VI, and the correlation coefficients and RRMSE were 0.89 and 23.4%, respectively (Table S4).

**Fig. 6.**
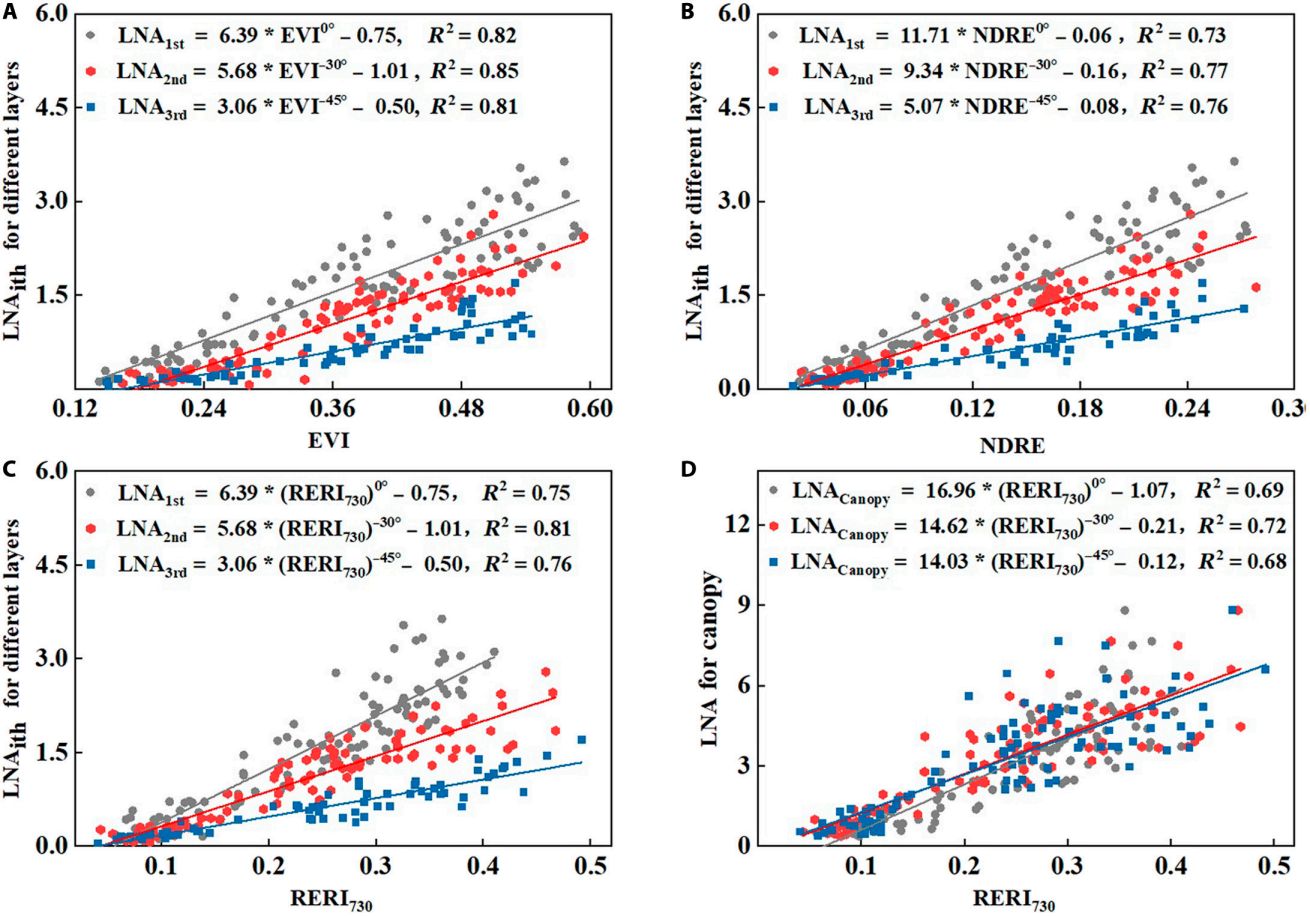
Linear relationship between different VIs and LNA: EVI and stratified LNA_ith_ (A), NDRE and stratified LNA_ith_ (B), RERI_730_ and stratified LNA_ith_ (C), and RERI_730_ and LNA_Canopy_ (D).

The optimal VIs (EVI, RERI_730_, and NDRE) for estimating LNA_ith_ in the linear model of each leaf layer are shown in Fig. 6. The slope (*k*) of the LNA_ith_ linear model in different leaf layers decreased sequentially, showing marked hierarchical differences. For example, the slope *k* of the RERI_730_ model in different leaf layers (LNA_1st_, LNA_2nd_, and LNA_3rd_) was 6.39, 5.68, and 3.06, respectively. In the nonstratified LNA_Canopy_ estimation model, although there were differences in the slope at different VZAs (*k* was 16.96, 14.62, and 14.03, respectively), the distribution was relatively consistent, especially in the middle and lower leaf layers.

### Comparison of accuracy on different LNA estimation models

#### Comparison on accuracy of different LNA estimation models based on linear regression

Based on the optimal models of LNA_ith_ (Fig. 6) and the relationship between LNA_ith_ and LNA_Canopy_ in each leaf layer (Table 1), LNA_Canopy_ estimation models considering the vertical heterogeneity of wheat were constructed. The validation accuracy of each model based on measured UAV data is shown in Table 2. The LNA_Canopy_ estimation model (LNA_sum_), which combined the leaf layers of LNA_1st_, LNA_2nd_, and LNA_3rd_, exhibited the highest accuracy, achieving an RRMSE of 19.3%. The next was the model that used cumulative leaf layer LNA_4th_, with an RRMSE of 19.8%. In addition, the RRMSE of the LNA_Canopy_ estimation model using only LNA_2nd_ was 21.5%. The LNA_Canopy_ estimation models based on LNA_1st_ and LNA_5th_ performed poorly, with RRMSE values of 25.8% and 28.7%, respectively. An RRMSE of 31.2% was observed for the model centered on LNA_3rd_, indicating its lowest accuracy among all models evaluated in the study.

**Table 2.** The LNA_Canopy_ estimation model considering vertical heterogeneity within wheat canopy

Layers	Linear models for estimating LNA_Canopy_	RRMSE
**LNA**_**1st**_	LNA_Canopy_ = 8.75 * EVI^0°^ − 0.09	25.8%
**LNA**_**2nd**_	LNA_Canopy_ = 13.29 * RERI_730_^−30°^ − 1.88	21.5%
**LNA**_**3rd**_	LNA_Canopy_ = 18.30 * NDRE^−45°^ + 0.62	31.2%
**LNA**_**4th**_	LNA_Canopy_ = 8.75 * EVI^0°^ + 13.29 * RERI_730_^−30°^ − 1.97	19.8%
**LNA**_**5th**_	LNA_Canopy_ = 13.29 * RERI_730_^−30°^ + 18.30 * NDRE^−45°^ − 1.26	28.7%
**LNA**_**Sum**_	LNA_Canopy_ = 16.04 * NDRE^0°^+ 21.86 * NDRE^−30°^+ 18.30 * NDRE^−45°^ + 1.76	19.3%

**Fig. 7.**
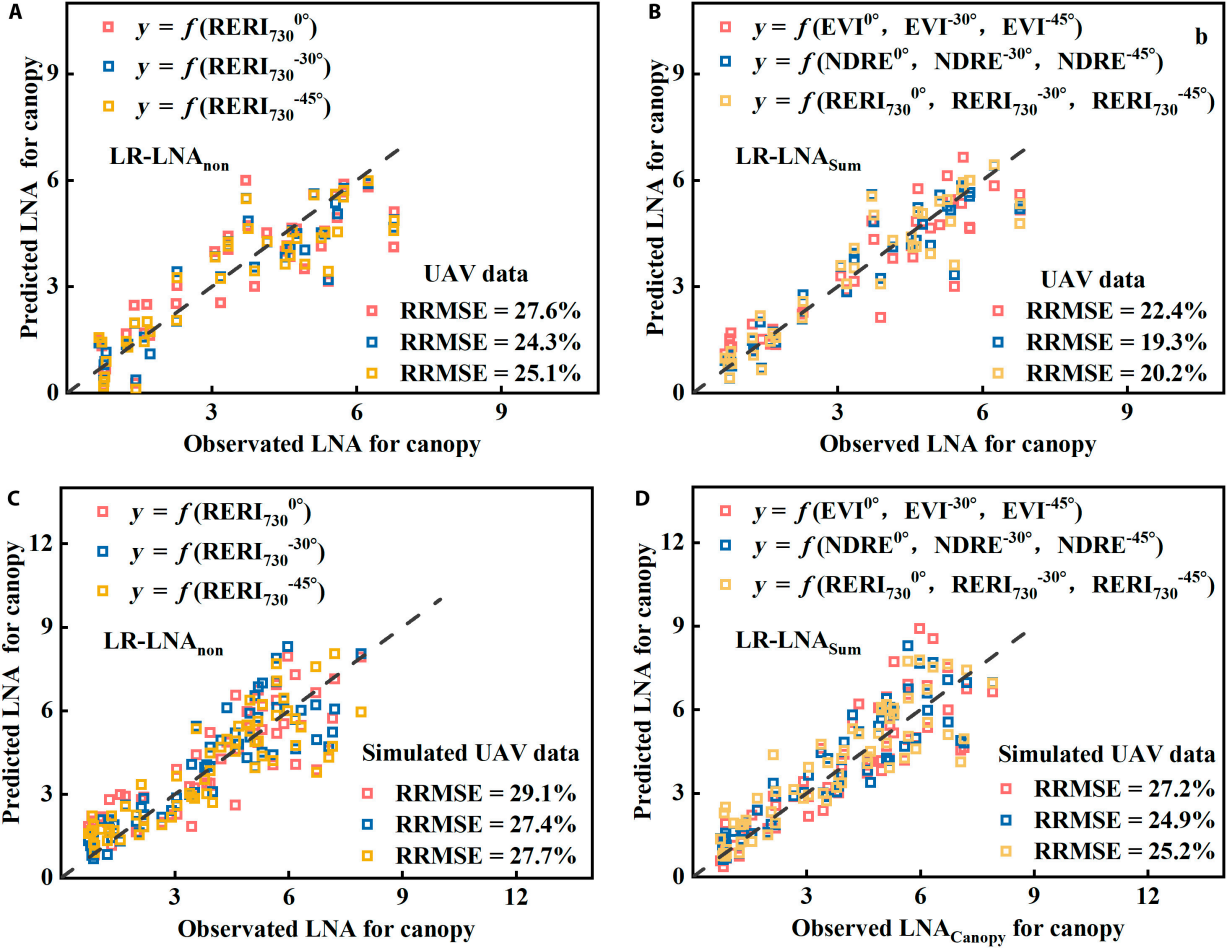
Accuracy validation of different linear LNA_Canopy_ estimation models: LR-LNA_non_ model (A) and LR-LNA_Sum_ model (B) for measured UAV data, and LR-LNA_non_ model (C) and LR-LNA_Sum_ model (D) for simulated UAV data.

Different VIs (EVI, NDRE, and RERI_730_) in the linear model (LR-LNA_Sum_) considering the accumulation of LNA in 3 layers were compared with the nonstratified LNA_Canopy_ linear model (LR-LNA_non_). The performance of the UAV measured dataset and simulated datasets is shown in Fig. 7. The results showed that the LR-LNA_non_ model based on the RERI_730_ index had the highest accuracy at a VZA of −30°, with RRMSE values of 24.3% and 27.4% for the UAV measured and simulated datasets, respectively. Compared with the LR-LNA_non_ model, the LR-LNA_Sum_ model was more accurate in estimating LNA_Canopy_. Among them, the LR-LNA_Sum_ model based on NDRE had the highest accuracy, with RRMSE values of 19.3% and 24.9% for UAV measured and simulated datasets, respectively. The accuracy of the LR-LNA_Sum_ model based on RERI_730_ was slightly lower than NDRE, with RRMSE values of 20.2% and 25.2% for UAV measured and simulated datasets, respectively. The LR-LNA_Sum_ model based on EVI had the lowest accuracy, with RRMSE values of 22.4% and 27.2% for UAV measured and simulated datasets, respectively.

#### Comparison on accuracy of different LNA estimation models based on the RF algorithm

**Fig. 8.**
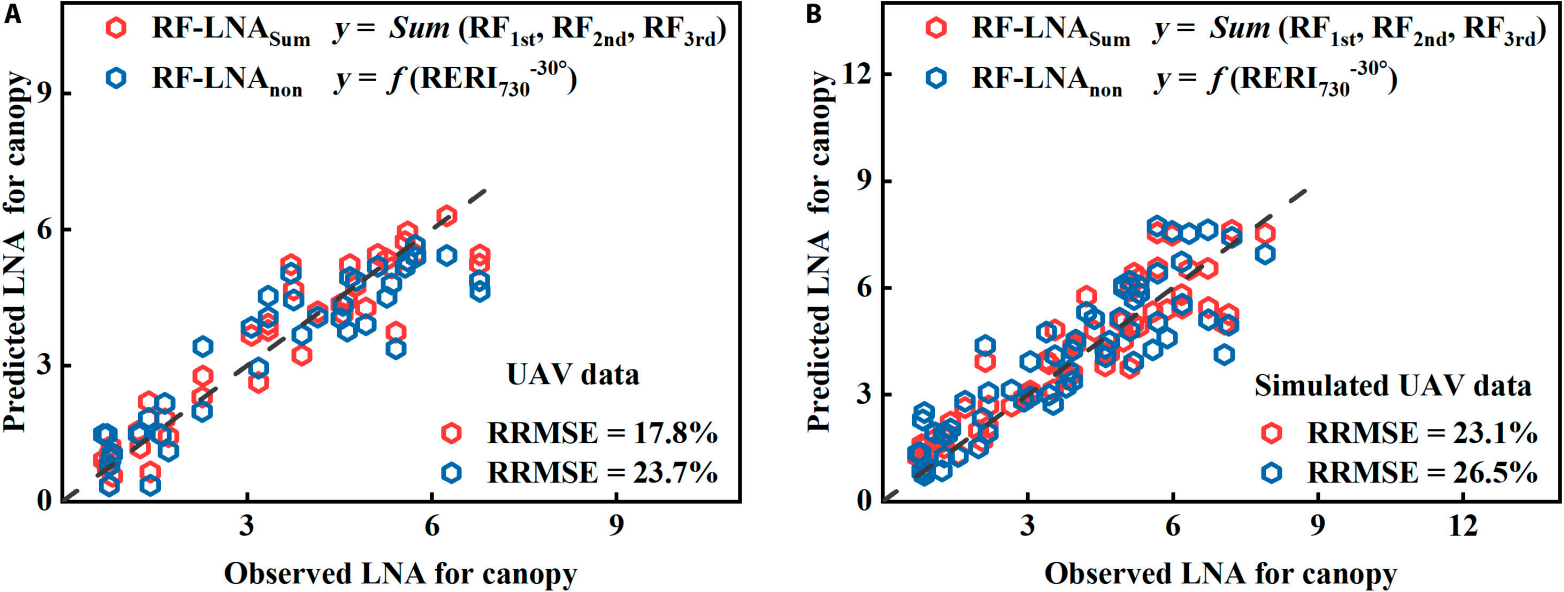
Validation accuracy of the LNA_Canopy_ estimation model based on RF regression: RF-LNA_Sum_ and RF-LNA_non_ model of measured UAV data (A) and RF-LNA_Sum_ and RF-LNA_non_ model accuracy of simulated UAV data (B).

To construct the LNA_Canopy_ estimation model based on the RF regression algorithm, the optimal indices of the LR-LNA_Sum_ and LR-LNA_non_ models at different VZAs were used as input parameters for the RF-LNA_Sum_ and RF-LNA_non_ models, respectively. The results (Fig. 8) showed that the RRMSE values of measured and simulated datasets of the unstratified RF-LNA_non_ model were 23.7% and 26.5%, respectively, while the accuracy of the RF-LNA_Sum_ model, considering vertical heterogeneity, was markedly higher than that of the unstratified RF-LNA_non_ model. The RRMSE values of the UAV measured and simulated datasets in the RF-LNA_Sum_ model were 17.8% and 23.1%, respectively.

#### Evaluation and validation on different LNA_Canopy_ estimation models based on field-scale data

The model validation results based on the UAV simulated dataset of the field-scale experiments are shown in Fig. S4. The results demonstrated that the LR-LNA_Sum_ model, considering vertical heterogeneity, achieved an *R*^2^ and RRMSE of 0.81 and 25.6%, respectively, outperforming the unstratified LR-LNA_non_ model (*R*^2^ and RRMSE were 0.78 and 29.8%, respectively). The RF-LNA_Sum_ and RF-LNA_non_ models showed higher accuracies than the linear regression mode (LR-LNA). The *R*^2^ and RRMSE of the RF-LNA_Sum_ model considering vertical heterogeneity were 0.88 and 21.4%, respectively. In comparison, the RF-LNA_non_ model had an *R*^2^ and RRMSE of 0.87 and 24.2%, respectively.

## Discussion

### Difference on vertical distribution of agronomic parameters in different leaf layers

In general, the LNC_ith_, LDW_ith_, and LNA_ith_ in different leaf layers exhibited similar variation characteristics to the corresponding canopy agronomic parameters (LNC_Canopy_, LDW_Canopy_, and LNA_Canopy_) throughout the growth period. LNC_ith_ generally decreased from the top to the bottom of the canopy (Fig. 4), which was consistent with previous studies [3,28]. This vertical distribution pattern may be attributed to changes in canopy structure during the growth period of crops. These changes result in marked differences in light interception by the crop canopy in the vertical direction, ultimately leading to vertical heterogeneity in physiological parameters such as nitrogen [42]. Currently, theories about vertical distribution of crop canopy are mainly divided into synergistic theory [43] and optimization theory [44]. The synergistic theory suggests that crops preferentially allocate nitrogen to the upper leaves with high light use efficiency. In contrast, the optimization theory posits that the relationship between canopy nitrogen distribution and leaf light absorption is the result of rebalancing. According to both theories, the vertical distribution of canopy physiological parameters is an inherent characteristic of crops and is less affected by crop varieties and environmental conditions [27]. Furthermore, canopy structure determines the distribution of light within the vegetation canopy and is also an important factor affecting the vertical distribution of crop nitrogen [45].

Obvious differences were observed between LNC_4th_, LNC_5th_, and LNC_Canopy_ at low nitrogen levels during the late growth period. This difference is primarily due to the transfer of nitrogen from the lower, older leaves to the newer leaves in the upper layer when nitrogen is deficient [1]. As a result, the nitrogen concentration in the lower leaves is lower than that in the middle and upper layers [29]. LNA_ith_ and LDW_ith_ in different layers initially increased and then decreased with the growth stage of wheat. However, the difference in LNA_ith_ between high nitrogen levels was much greater than that at low nitrogen levels. This is mainly because crops with high nitrogen levels are more nutritionally adequate and have a larger LDW accumulation, and LNA is mainly affected by LDW under the same nitrogen level.

### Effects of vertical heterogeneity on the accuracy of wheat canopy LNA estimation

The canopy of crops has often been regarded as a uniform medium, and the entire nutrient status of crops has traditionally been reflected by single-point spectral information. However, due to the vertical heterogeneity of crop growth, the estimation accuracy of crop nitrogen status varies when using different leaf layers [3]. In this study, the accuracy of the LNA_Canopy_ estimation model based on LNA_2nd_ was higher than that based on LNA_1st_ and LNA_3rd_ (Tables 1 and 2). The main reason for this may be that the upper layer receives sufficient light, and the nitrogen supply priority of the upper young leaves is higher. As a result, even under nitrogen stress conditions, the upper leaves can maintain a higher nitrogen content, thereby maximizing photosynthetic efficiency. Therefore, it is difficult for the upper leaves to reflect the whole picture of crop nitrogen status [7]. On the other hand, the middle–upper leaves are generally fully developed, and their biomass and dry weight are relatively stable, making them closely related to the canopy leaf biomass [3]. Therefore, the estimation accuracies of LNA_Canopy_ through LNA_Sum_ and LNA_4th_ are higher than those of the unstratified model (LNA_non_). In contrast, the lower layers had the lowest accuracy due to the abundance of yellow leaves, low nitrogen content, and dry weight of leaves (Tables 1 and 2).

In addition, the sunlight also has a certain impact on the accuracy of the model, mainly due to the influence of changes in the position of the sun on the spectral reflectivity. When the relative position of the light and drone sensor’s field of view changes, the crop soil shadow ratio within the field of view also changes accordingly, which would affect the model accuracy. For instance, when the light is consistent with the field of view of the drone sensor, the proportion of crops in the field of view is the highest, and the proportion of soil and shadows is relatively low. At this point, the measured crop reflectance is minimally affected by soil and shadows, and the accuracy of the model is relatively high [46]. Although this study attempted to maintain consistency for the time of each data collection, it is still difficult to avoid the impact of solar position changes on model accuracy. Moreover, other environmental conditions like wind speed, sunshine intensity, precipitation, and temperature may also affect the vertical heterogeneity of crops, and coupling remote sensing data and crop growth models should be a potential solution to further improve the modeling accuracy [47].

### Potential of the LNA_Sum_ model considering vertical heterogeneity

Previous researchers have often used vertical canopy reflectance or VIs to estimate LNA, but rarely considered the effect of vertical nitrogen heterogeneity [48]. The accuracy of monitoring targets is markedly affected by the substantial differences in the contribution of spectral reflectance from different leaf layers [30,49]. Previous studies have shown that the vertical observation direction mainly collected spectral information from the upper leaves; thus, the estimation accuracy of nitrogen indices for the upper, middle, and lower layers of crops would decrease obviously when using reflectance data collected in the nadir direction [3,7]. The results of this study showed that the LNA estimation model constructed by combining multiple VZAs could effectively reflect the differences in spectral information among different leaf layers of crops in time. For example, using reflectance at vertical VZA could have a higher estimation accuracy of LNA in the upper leaf layer (Fig. 5). This may be because the experiment in this study began in the middle of the jointing stage, and the influence of soil background was relatively small. Therefore, when estimating LNA in the upper layer, the advantage of tilt VZAs was not particularly prominent. However, it is more accurate to monitor LNA in the middle and lower layers using oblique observation VZAs (such as −30° and −45°) (Fig. 5). This may be because the symptoms of nitrogen deficiency are mainly manifested in the middle and lower layers when nitrogen deficiency is mild to moderate. During this period, the deficiency state of nitrogen in the middle and lower layers can be captured more promptly using oblique observation methods [29].

The optimal monitoring VIs for the upper, middle, and lower layers was EVI, RERI_730_, and NDRE, respectively (Fig. 6). This is because visible and near-infrared bands mainly affect the characteristics of leaves in the upper layer, while VIs incorporating the red edge band have greater penetration into the canopy, making them more suitable for monitoring nitrogen status in the middle and lower layers [30,31]. Additionally, the difference of slope *k* in the linear relationship between the LNC_ith_, LNA_ith_, and LDW_ith_ in different leaf layers and the VIs at different VZAs (Fig. 6) also indicates that multiangular observation can better capture the vertical allometry of wheat. Therefore, it is necessary to consider vertical heterogeneity when estimating physiological parameters of crops [50].

In this study, different combination schemes were optimized by analyzing the optimal VZAs and suitable monitoring indices of each leaf layer (Table 2). If researchers only need to monitor the LNA status in the upper leaf layer, the EVI model at nadir direction could be employed (RRMSE of 25.8%). Similarly, only RERI_730_ or NDRE models at oblique VZAs (−30° or −45°) can be used to estimate wheat LNA in the middle or lower layers (RRMSE was 21.5% and 31.2%, respectively). If more accurate LNA estimation results were required, this study recommends using the LNA_Sum_ model, which considers the upper, middle, and lower layers simultaneously (RRMSE was 19.3%).

Despite the potential for accumulated errors when combining multiple VZAs and different layers to estimate canopy LNA, the estimation accuracy of the LNA_Sum_ model, considering the influence of vertical heterogeneity, was still higher (Figs. 7 and 8 and Fig. S4). Since machine learning offers better stability and stronger portability, the accuracy of LNA estimation at each layer using RF regression was better than that of the linear model. In addition, the VZA effect was another factor considered in this study when using multiangular remote sensing to estimate LNA. RERI_730_ and NDRE were generally less affected by the VZA effect (Fig. S3). This may be another reason why they had higher accuracy to estimate LNA than other VIs. Finally, the model established in this study was based on the measured UAV data, and the UAV data simulated by ASD still differed from measured UAV data, including the following 3 aspects: (a) the differences in sensors between the two, (b) the difficulty in ensuring absolute consistency in the proportion of crops within the field of view of UAV and ASD data during the data collection process, and (c) the difficulty in ensuring absolute synchronization of data collection time between the two. Therefore, the estimation accuracy of the UAV-simulated dataset was lower than that of the UAV-measured dataset (Figs. 7 and 8). In a future study, the biases of the estimation accuracy between measured and simulated UAV data could be further corrected through the Bidirectional Reflectance Distribution Function model [35].

However, the stability of the LNA_Sum_ based on both linear and RF algorithm across different datasets and wheat varieties still proves its wide applicability. Although the LNA_Sum_ model was only for wheat crop in this study, some previous studies have analyzed the vertical distribution characteristics of other crops, such as rice and maize [3,51], and the main morphological characteristic of these crops is the tall upright type, which is similar to wheat crop. Therefore, the LNA_sum_ model has great potential to be expanded to these crops, yet the hierarchical estimation approach may not be applicable to crops with vines or short stems. Also, field experiments with more crop varieties, N treatments, and climate conditions are needed to further expand the utility of the model.

## Conclusion

In this study, the optimal LNA_ith_ estimation models for different leaf layers were constructed based on the vertical heterogeneity characteristics of crop growth, and the advantages of vertical and multiangular remote sensing were comprehensively utilized to construct estimation models of canopy LNA through the optimal LNA_ith_ models of single leaf layers, namely, LR-LNA_Sum_ and RF-LNA_Sum_. The results showed that the changes of LNC_ith_, LNA_ith_, and LDW_ith_ of wheat in different leaf layers were consistent with those of canopy LNC, LNA, and LDW. LNC_ith_ decreased with the growth period and also decreased from top to bottom in the vertical distribution direction. Both LNA_ith_ and LDW_ith_ increased first and then decreased with the growth period. At low nitrogen levels, the differences among LNC_Canopy_, LNC_4th_, and LNC_5th_ increased markedly with the growth stages. The optimum VZAs, monitoring bands, and VIs of LNA_ith_ in different leaf layers were obviously different. For the LNA_1st_ layer, the optimum VZA, monitoring band, and VI were 0°, near-infrared band, and EVI, respectively. For the middle and lower layers, they were −30° or −45°, red-edge band with strong penetrating ability, and RERI_730_ and NDRE, respectively. The accuracy of LNA_Canopy_ estimation based on the combination of LNA_ith_ from different leaf layers also varied, with the model based on cumulative 3 layers (LNA_Sum_) showing the highest accuracy. The slope *k* showed an obvious stratification effect in the linear relationship between the LNA_ith_ of different leaf layers and the same VI at different VZAs. The accuracy of LR-LNA_Sum_ and RF-LNA_Sum_ models, considering the effect of vertical heterogeneity under different datasets and wheat varieties, was higher than that of unstratified LR-LNA_Sum_ and RF-LNA_Sum_ models. This finding could provide technical support for remote sensing estimation of crop canopy growth parameters.

## Data Availability

All data supporting the findings of this study are available within the article.
