# Supplementary material for: Estimating Leaf Nitrogen Accumulation Considering Vertical Heterogeneity Using Multiangular Unmanned Aerial Vehicle Remote Sensing in Wheat
Source: Plant Phenomics. 2024 Dec 5;6:0276. doi: 10.34133/plantphenomics.0276 (PMC11617620; doi:10.34133/plantphenomics.0276)
Supplement: Supplementary 1 — Figs. S1 to S4 Tables S1 to S4 [file plantphenomics.0276.f1.zip › Supplementary Material file10.24.docx]

**Supplementary Material file**

**Title:** Estimating leaf nitrogen accumulation considering vertical heterogeneity using multi-angular UAV remote sensing in wheat

**Yuanyuan Pan, ^1,2^ Jingyu Li, ^1^ Jiayi Zhang, ^1^ Jiaoyang He, ^1^ Zhihao Zhang, ^1^** **Xia Yao, ^1^ Tao Cheng, ^1^ Yan Zhu, ^1^ Weixing Cao, ^1^ Yongchao Tian ^1,^***

^1^ National Engineering and Technology Center for Information Agriculture, Engineering and Research Center of Smart Agriculture (Ministry of Education), Key Laboratory for Crop System Analysis and Decision Making (Ministry of Agriculture and Rural Affairs), Jiangsu Key Laboratory for Information Agriculture, Jiangsu Collaborative Innovation Center for Modern Crop Production, Nanjing Agricultural University, Nanjing, 210095, China.

^2^ Jiangsu Academy of Agricultural Sciences Wuxi Branch, Wuxi, 214174, China.

*****Correspondence: [yctian@njau.edu.cn](mailto:yctian@njau.edu.cn); Tel: +86–25–84399050; Fax: +86-25-84396672.

**This supplementary file includes:**

**Fig. S.1**

**Fig. S.2**

**Fig. S.3**

**Fig. S.4**

**Table S.1**

**Table S.2**

**Table S.3**

**Table S.4**

**Fig. S.1**


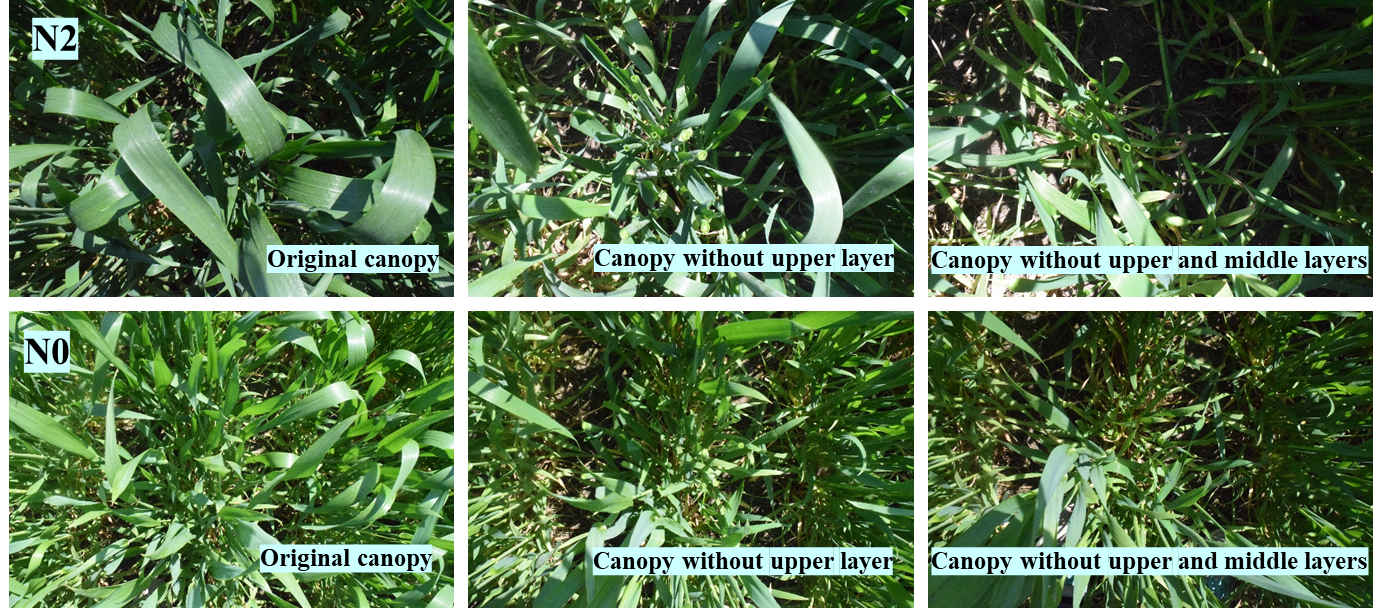


**Fig. S.1** Sampling display of different layers. Note: N0 and N2 represent nitrogen application rates of 0 and 300 kg N ha^-1^, respectively.

**Fig. S.2**


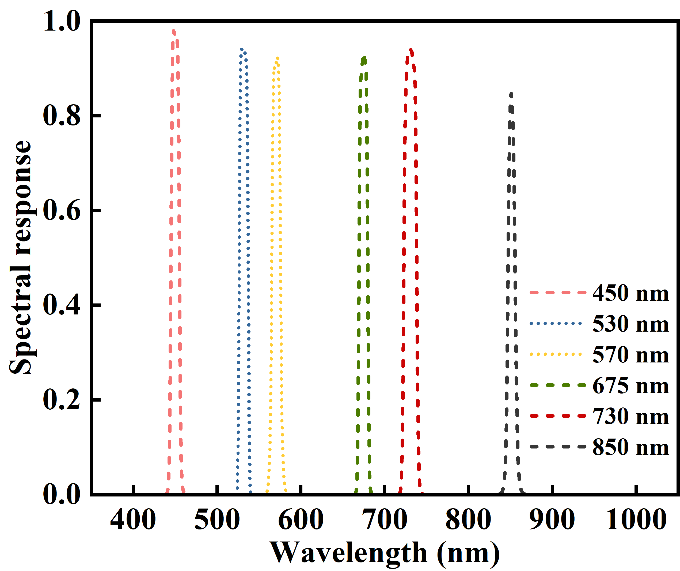


**Fig. S.2** The spectral response function of airphen optical channels.

**Fig. S.3**


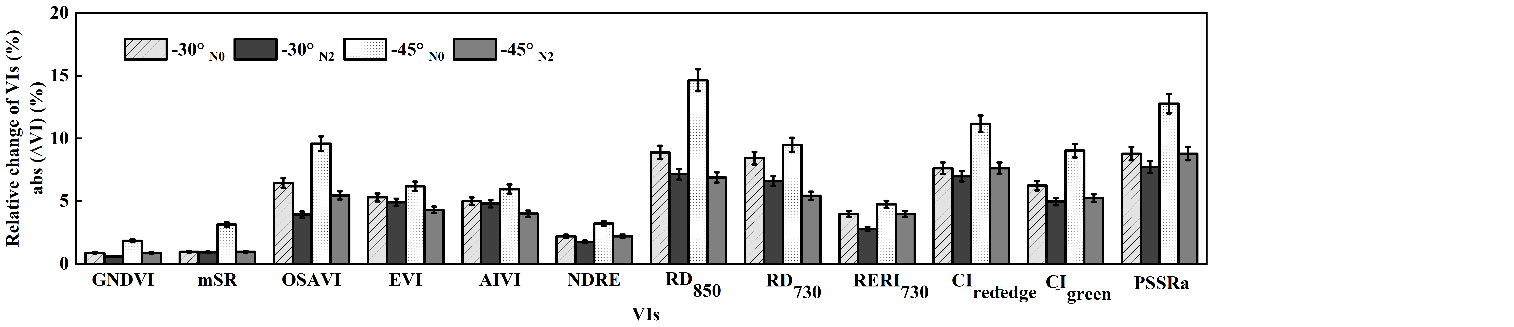


**Fig. S.3** Relative changes of VIs under different view zenith angles (VZAs).

**Fig. S.4**


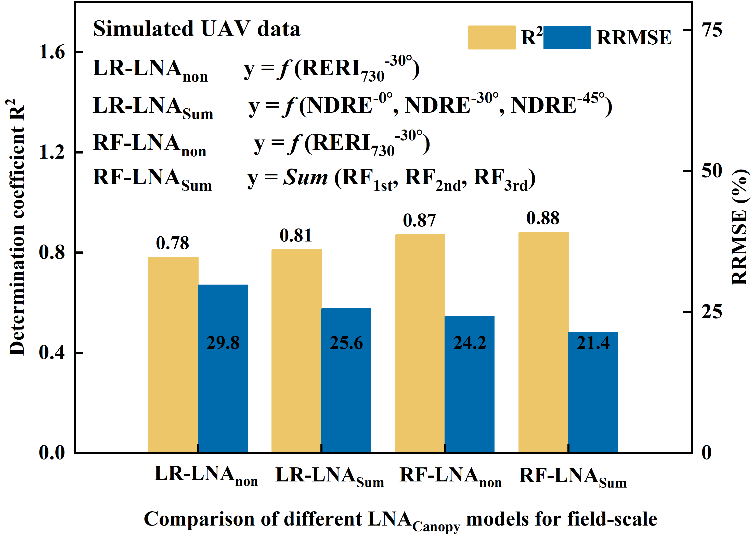


**Fig. S.4** Validation accuracy of different LNA_Canopy_ estimation models in field-scale experiments.

**Table S.1**

**Table S.1** Detailed information of wheat experiments conducted in 2018-2021.

| Exp. No | Year | Varieties | Sowing time | Treatment | Sampling stage |
| --- | --- | --- | --- | --- | --- |
| **Exp. 1** | 2018-2019 | SX 6, YM 16 | 28/10/2018 | N rate (kg ha^-1^): 0, 150, 300  Row spacing (cm): 25, 40 | Jointing, Booting, Heading, Flowering, Forward-filling, Mid-filling |
| **Exp.2** | 2020-2021 | SX 6, YM 16 | 27/10/2020 | N rate (kg ha^-1^): 0, 150, 300  Row spacing (cm): 30, 40 | Jointing, Booting, Heading, Flowering, Forward-filling, Mid-filling |
| **Exp.3** | 2020-2021 | YF 4, YF 101; SM 8; SX 6; YM 18, YM 20, YM 23, YM 25, YM 29; NM 13, NM 22; HM 5, HM 7; ZM 9, ZM 10, ZM 12, ZM 13, ZM 168; RH 596 | 05/11/2020 | N rate (kg ha^-1^): 150; 300  Row spacing (cm): 30 | Jointing, Booting |

**Note:** SX: shengxuan, YM: Yangmai, SM: Sumai, NM: Ningmai, HM: Huamai, ZM: Zhenmai, YF: Yangfu, RH: Ruihua.

**Table S.2**

**Table S.2.** Introduction of selected VIs in this study.

| **Index** | **Formulation** | | **Reference** |
| --- | --- | --- | --- |
| Green Normalized Different Vegetation Index (gNDVI) | | $\left( \rho_{\text{nir }}-\rho_{\text{green }} \right)/\left( \rho_{\text{nir }}+\rho_{\text{green }} \right)$ | (Gitelson et al., 1996) |
| Normalized Difference Red edge Index (NDRE) | | $\left( \rho_{\text{nir }}-\rho_{\text{rededge }} \right)/\left( \rho_{\text{nir }}+\rho_{\text{rededge }} \right)$ | (Cao et al., 2013) |
| Modified Red-edge Ratio Index (mSR) | | $\left( \rho_{\text{rededge}}-\rho_{\text{blue }} \right)/\left( \rho_{\text{rededge}}+\rho_{\text{blue }} \right)$ | (Sims and Gamon, 2002) |
| Red Difference Vegetation Index (RD_850_) | | $\rho_{\text{nir }}-\rho_{\text{red }}$ | (Tucker, 1979) |
| Red Edge Difference Vegetation Index (RD_730_) | | $\rho_{\text{rededge }}-\rho_{\text{green }}$ | (Tucker, 1979) |
| Red Edge Relative  Index (RERI_730_) | | $1-\rho_{\text{rededge }}/\rho_{\text{nir }}$ | (Xu et al., 2019) |
| Green Chlorophyll Index (CI_green_) | | $\rho_{\text{rededge }}/\rho_{\text{green }}-$1 | (Gitelson et al., 2003) |
| Red edge Chlorophyll Index (CI_rededge_) | | $\rho_{\text{nir }}/\rho_{\text{rededge }}-$1 | (Gitelson et al., 2003) |
| Pigment Specific Simple Ratio Index (PSSRa) | | $\rho_{\text{nir }}/\rho_{\text{red }}$ | (Blackburn, 1998) |
| Optimal Soil Adjusted Vegetation Index (OSAVI) | | $(1+L)\left( \rho_{\text{nir }}-\rho_{\text{red }} \right)/\left( \rho_{\text{nir }}+\rho_{\text{red }}+L \right)，L=0.16$ | (Xu et al., 2019) |
| Enhanced Vegetation Index (EVI) | | $2.5\left( \rho_{\mathrm{nir}}-\rho_{\mathrm{red}} \right)/(\rho_{\text{nir }}+6*\rho_{\text{red }}-7.5*\rho_{\mathrm{blue}}+1)$ | (Gitelson et al., 1996) |
| Angular Insensitivity Vegetation Index (AIVI) | | $(1+L)\left( \rho_{\text{nir }}-\rho_{\text{red }} \right)/\left( \rho_{\text{nir }}+\rho_{\text{red }}+L \right)，L=0.5$ | (Huete, 1988) |

Note: ρ was canopy reflectance. Bands in NIR, red-edge, red, blue were wavelengths at 850 nm, 730 nm, 675 nm, 450 nm, respectively.

**Table S.3**

| **Table S.3** The 10-fold cross validation results of RF models for predicting LNA based on different VZAs and VIs. | | | | | | |
| --- | --- | --- | --- | --- | --- | --- |
| VZA  (Layer) | EVI | | NDRE | | RERI_730_ | |
|  | *R^2^* | *RRMSE* | *R^2^* | *RRMSE* | *R^2^* | *RRMSE* |
| 0° (1st) | 0.79 | 17.8% | 0.76 | 18.6% | 0.76 | 18.3% |
| -30°(2nd) | 0.81 | 17.5% | 0.74 | 17.4% | 0.83 | 17.7% |
| -45° (3rd) | 0.78 | 19.1% | 0.79 | 16.8% | 0.81 | 19.2% |

**Table S.4**

**Table S.4.** The optical VIs, view zenith angles (VZAs) and estimation accuracy of each leaf layer.

| **Stratified layers** | **VIs** | **Optical VZAs** | **R** | **RRMSE** |
| --- | --- | --- | --- | --- |
| **LNA_1st_** | EVI | 0° | 0.88 | 18.3% |
| **LNA_2nd_** | RERI_730_ | -30° | 0.90 | 18.1% |
| **LNA_3rd_** | NDRE | -45° | 0.91 | 18.4% |
| **LNA_Canopy_** | RERI_730_ | -30° | 0.89 | 23.4% |

**References**

1. Blackburn, G.A., 1998. Quantifying chlorophylls and caroteniods at leaf and canopy scales: an evaluation of some hyperspectral approaches. Remote Sens. Environ. 66, 273-285. http://doi.org/10.1016/S0034-4257(98)00059-5.
2. Cao, Q., Miao, Y., Wang, H., Huang, S., Cheng, S., Khosla, R., Jiang, R., 2013. Non-destructive estimation of rice plant nitrogen status with crop circle multispectral active canopy sensor. Field Crop. Res. 154, 133-144. http://doi.org/10.1016/j.fcr.2013.08.005.
3. Gitelson, A.A., Gritz, Y., Merzlyak, M.N., 2003. Relationships between leaf chlorophyll content and spectral reflectance and algorithms for non-destructive chlorophyll assessment in higher plant leaves. J. Plant Physiol. 160, 271-282. http://doi.org/10.1078/0176-1617-00887.
4. Gitelson, A.A., Kaufman, Y.J., Merzlyak, M.N., 1996. Use of a green channel in remote sensing of global vegetation from eos-modis. Remote Sens. Environ. 58, 289-298. http://doi.org/10.1016/S0034-4257(96)00072-7.
5. Huete, A.R., 1988. A soil-adjusted vegetation index (savi). Remote Sens. Environ. 25, 295-309. http://doi.org/10.1016/0034-4257(88)90106-X.
6. Sims, D.A., Gamon, J.A., 2002. Relationships between leaf pigment content and spectral reflectance across a wide range of species, leaf structures and developmental stages. Remote Sens. Environ. 81, 337-354. https://doi.org/10.1016/S0034-4257(02)00010-X.
7. Tucker, C.J., 1979. Red and photographic infrared linear combinations for monitoring vegetation. Remote Sens. Environ. 8, 127-150. https://doi.org/10.1016/0034-4257(79)90013-0.
8. Xu, M., Liu, R., Chen, J.M., Liu, Y., Shang, R., Ju, W., Wu, C., Huang, W., 2019. Retrieving leaf chlorophyll content using a matrix-based vegetation index combination approach. Remote Sens. Environ. 224, 60-73. https://doi.org/10.1016/j.rse.2019.01.039.
