# Supplementary figures and images for: Estimating Leaf Nitrogen Accumulation Considering Vertical Heterogeneity Using Multiangular Unmanned Aerial Vehicle Remote Sensing in Wheat
Source: Plant Phenomics. 2024 Dec 5;6:0276. doi: 10.34133/plantphenomics.0276 (PMC11617620; doi:10.34133/plantphenomics.0276)

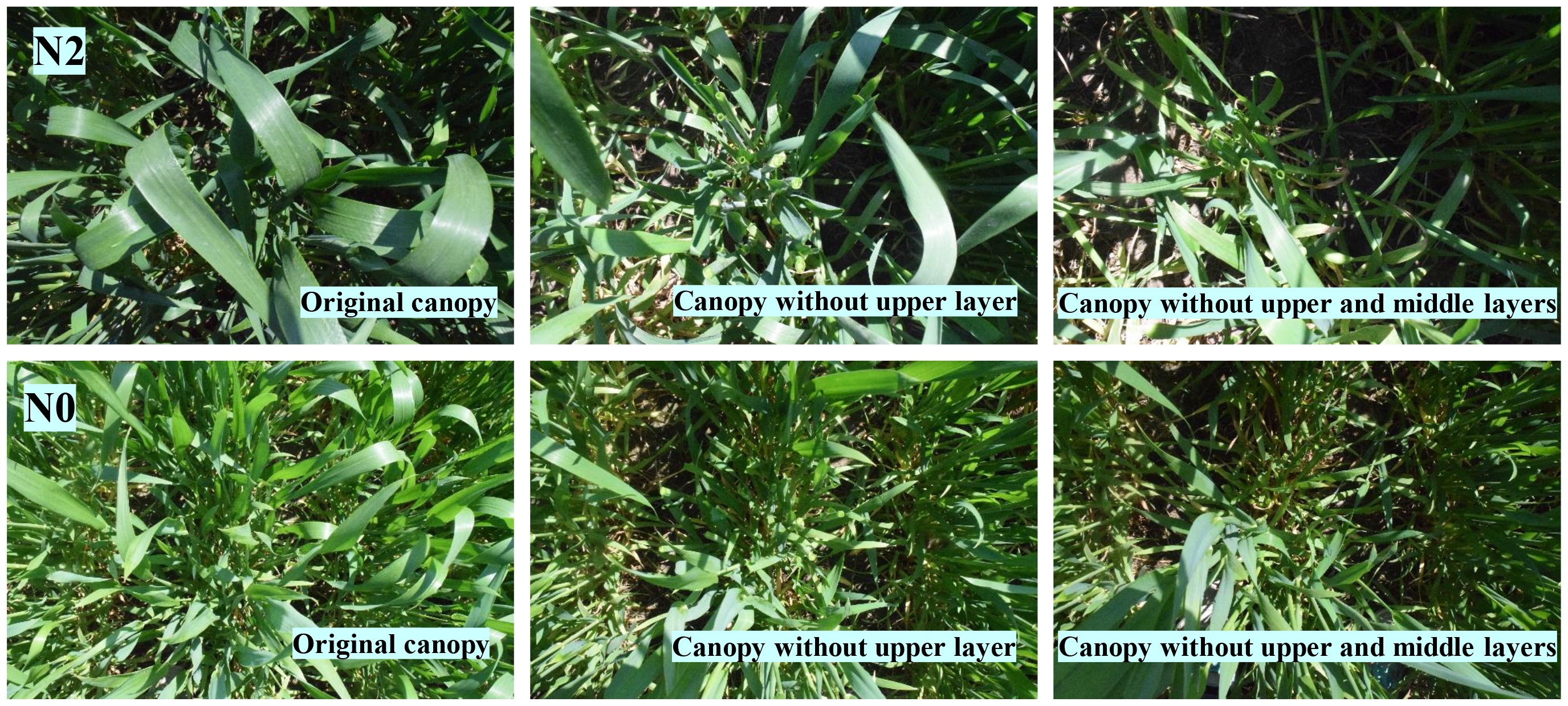

Supplement: Supplementary 1 — Figs. S1 to S4 Tables S1 to S4 [file plantphenomics.0276.f1.zip › Fig.S.1.tif]

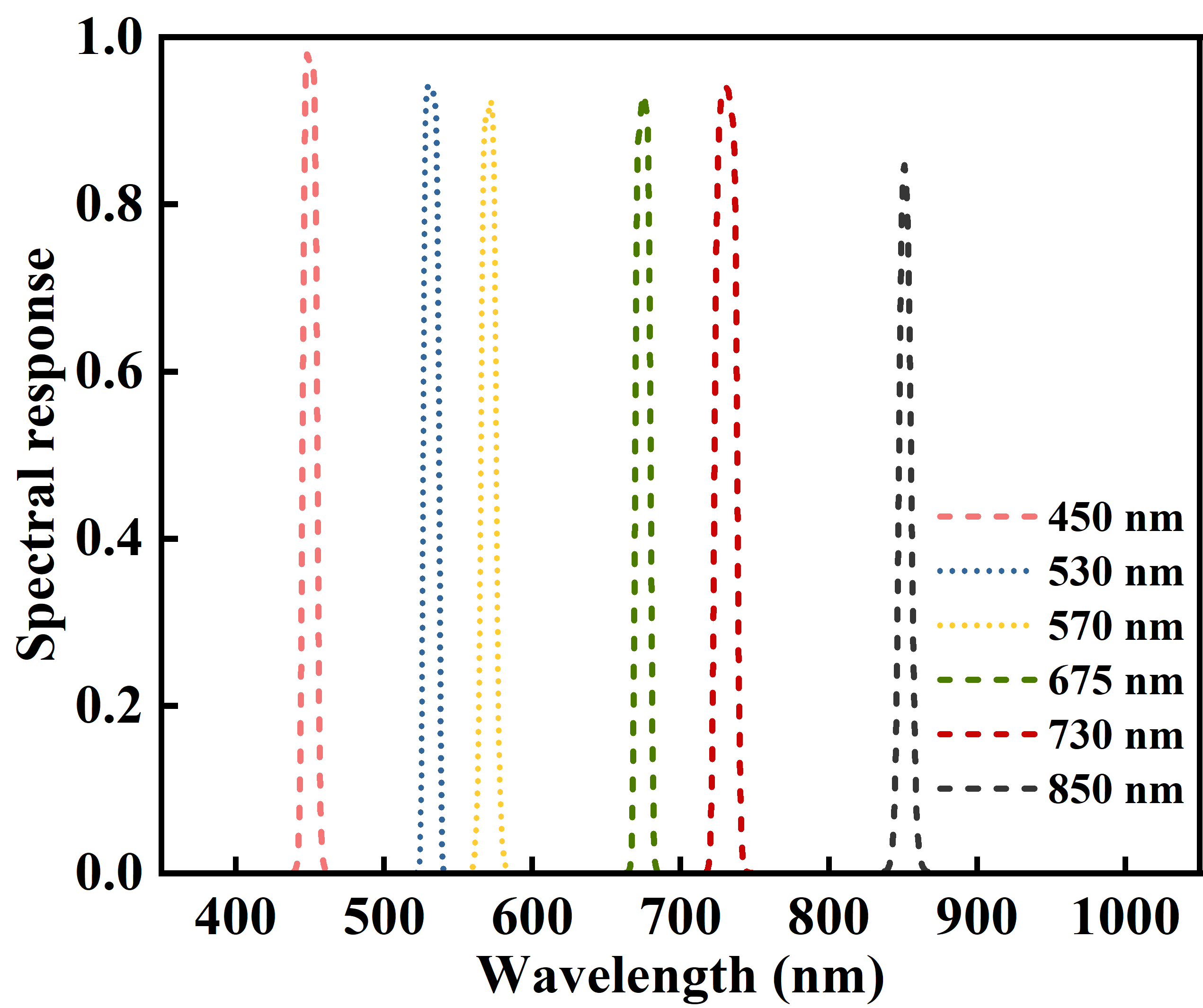

Supplement: Supplementary 1 — Figs. S1 to S4 Tables S1 to S4 [file plantphenomics.0276.f1.zip › Fig.S.2.tif]

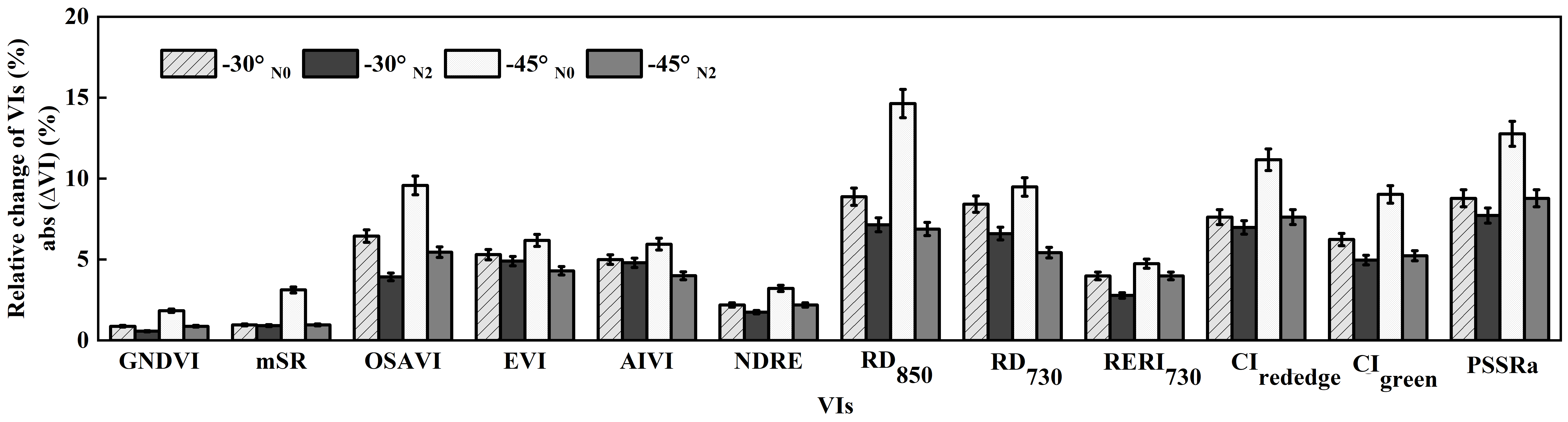

Supplement: Supplementary 1 — Figs. S1 to S4 Tables S1 to S4 [file plantphenomics.0276.f1.zip › Fig.S.3.tif]

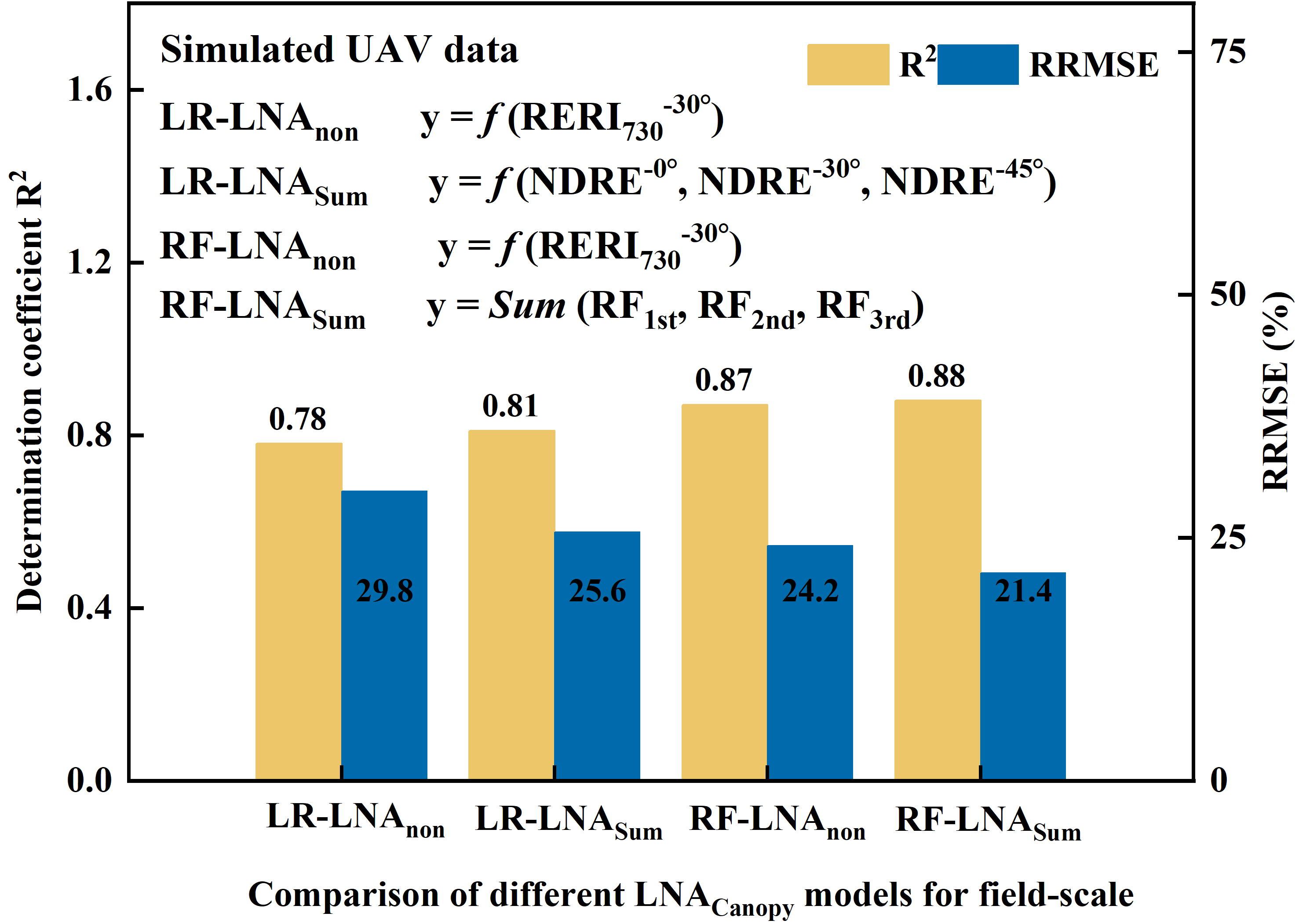

Supplement: Supplementary 1 — Figs. S1 to S4 Tables S1 to S4 [file plantphenomics.0276.f1.zip › Fig.S.4.tif]
